# Influence of Third Particle on the Tribological Behaviors of Diamond-like Carbon Films

**DOI:** 10.1038/srep38279

**Published:** 2016-12-05

**Authors:** Lichun Bai, Narasimalu Srikanth, Guozheng Kang, Kun Zhou

**Affiliations:** 1 Interdisciplinary Graduate School, Nanyang Technological University, 50 Nanyang Avenue, Singapore 639798, Singapore; 2 School of Mechanical and Aerospace Engineering, Nanyang Technological University, 50 Nanyang Avenue, Singapore 639798, Singapore; 3 Applied Mechanics and Structure Safety Key Laboratory of Sichuan Province, School of Mechanics and Engineering, Southwest Jiaotong University, Chengdu, Sichuan 610031, China

## Abstract

Tribological mechanisms of diamond-like carbon (DLC) films in a sand-dust environment are commonly unclear due to the complicated three-body abrasion caused by sand particles. This study investigates the three-body abrasion of the DLC film via molecular dynamics simulations. The influence factors such as the load, velocity, shape of the particle and its size are considered. It has been found that the friction and wear of the DLC film are determined by adhesion at a small load but dominated by both adhesion and plowing at a large load. A high velocity can increase the friction of the DLC film but decrease its wear, due to the response of its networks to a high strain rate indicated by such velocity. The shape of the particle highly affects its movement mode and thus changes the friction and wear of the DLC film. It is found that a small-sized particle can increase the friction and wear of the DLC film by enhancing plowing. These unique tribological mechanisms of the DLC film can help to promote its wide applications in a sand-dust environment.

Diamond-like carbon (DLC) films that consist of sp^2^ and sp^3^ hybridized carbon atoms exhibit excellent tribological behaviors^1^. The DLC film can highly reduce the friction and wear of workpieces in various environments such as water, desert and outer-space^2^. The sand or dust particles in the desert and outer-space can cause a three-body abrasion of the DLC film that is different from its commonly tribological mechanisms. Since the world is becoming desertization and more explorations are conducted in the outer-space this century, it is important to understand the tribological mechanisms of the DLC film to improve the stability of machines with the presence of sand or dust particles.

The mechanism of the three-body abrasion is complicated, because it is influenced by many factors such as the load, velocity, size of the particle, its shape and number density, hardness ratio of particle to substrates and hardness ratio between the substrates^3^. Moreover, the mechanical properties of DLC films vary largely according to their deposition parameters and compositions^4^, and thus further complicate their abrasion mechanisms.

Tribological behaviors of DLC films in a sand dust environment have been only studied in several experiments. Previous studies mainly located a large number of sand particles at the contact interface between the DLC films^5^,^6^,^7^,^8^. In fact, in common situations the surface damages of the DLC films are caused by few sand particles instead of a plenty of them. This is because the few particles can cause a huge contact stress and severely damage the DLC film by inducing its plastic deformation, thus degrading its surface morphology and structure and highly influencing its tribologcial behaviors. Influence of such few particles is hardly investigated in experiment. This is because these particles and the thickness of the DLC films are commonly at the microscale or nanoscale and the corresponding friction and wear phenomenon are hardly observed in experiment^4^.

In this case, simulations are commonly employed to understand the three-body abrasion. Zhang *et al*. reported that the substrate deformation in a three-body contact condition follows the regimes of no-wear, condensing, adhering and ploughing^9^. Sun *et al*. found that the nanoparticle purely rolls during the friction process and the wear of materials is dominated by plowing^10^. In the study by Si *et al*., wear caused by the particle rolling is high and cannot be neglected compared with that by sliding^11^. These previous simulations provide a fundamental for the understanding of the three-body abrasion at the nanoscale.

This study investigates the tribological behaviors of DLC films in a three-body contact condition via molecular dynamics (MD) simulations. The influence factors such as the load, velocity, shape of the particle and its size are considered. It is believed that this study can improve the understanding of the three-body abrasive mechanisms of the DLC film and promote its applications in a sand-dust environment.

## Results and Discussions

### Load effect

**Figure 1:**
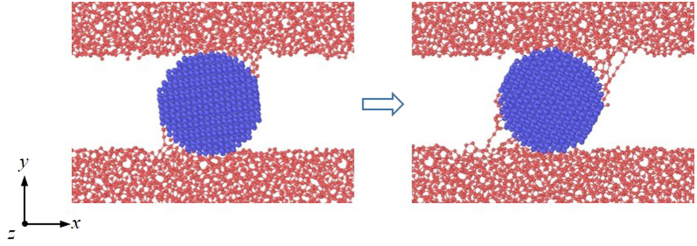
Atomic configuration of the friction process with a spherical particle.

The load effect is considered in the cases with a spherical particle. The sliding configuration in Fig. 1 shows that many covalent bonds are formed at the interface between the particle and the DLC films, indicating that the sticky DLC networks attach to the spherical particle. These bonds cause a strong interfacial adhesion and thus influence the movement of the particle that can be evaluated by calculating its mass-center velocity. It is found that the spherical particles undergo pure rolling in the friction process. This is consistent with observations in the literature^10^. The sliding configuration demonstrates that the friction and wear of the DLC films with a rolling third particle may closely relate with adhesion.

**Figure 2:**
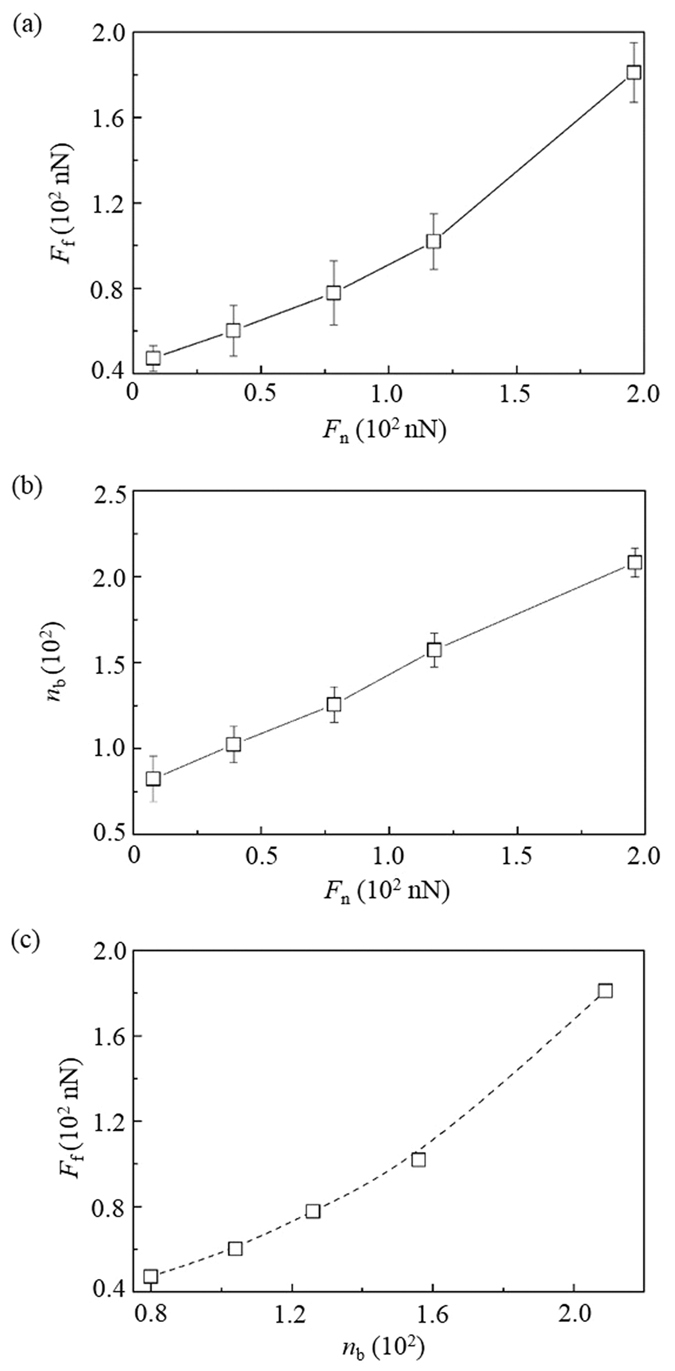
Effect of load *F*_n_ of the DLC film on (**a**) its friction force *F*_f_ and (**b**) the number of contact bonds *n*_b_; (**c**) *F*_f_ vs *n*_b_.

When the load *F*_n_ increases, the friction force *F*_f_ of the DLC films increases (Fig. 2a). The increase of *F*_f_ is because a large *F*_n_ can penetrate the particle into the DLC films and thus increase the interfacial adhesion strength. This can be verified by the increase of the number of bonds *n*_b_ at the contact interface with the *F*_n_ (Fig. 2b). The *n*_b_ is the total number of bonds formed between the particle and the DLC films, due to the fact that all these bonds contribute to the interfacial adhesion.

Recent theoretical studies showed that at the nanoscale *F*_f_ is linearly proportional to *n*_b_ which represents the real contact area, indicating the validation of the macroscale Bowden-Tabor model at the nanoscale^12^,^13^. The configuration in Fig. 1 has shown that the friction and wear highly depend on the interfacial adhesion due to the pure rolling of the particle. Therefore, the *F*_f_ in the three-body contact condition should also be closely related with *n*_b_. In this case the relation between the *F*_f_ and *n*_b_ in the three-body contact condition is firstly studied, as shown in Fig. 2c. It can be seen that this relation can be regarded as linear with a small *n*_b_ but becomes nonlinear when the *n*_b_ is large. Such relation evolution indicates that at a small load *F*_n_ the friction is simply determined by the interfacial adhesion strength while at a large *F*_n_ the friction is dominated by both the adhesion and other factors.

**Figure 3:**
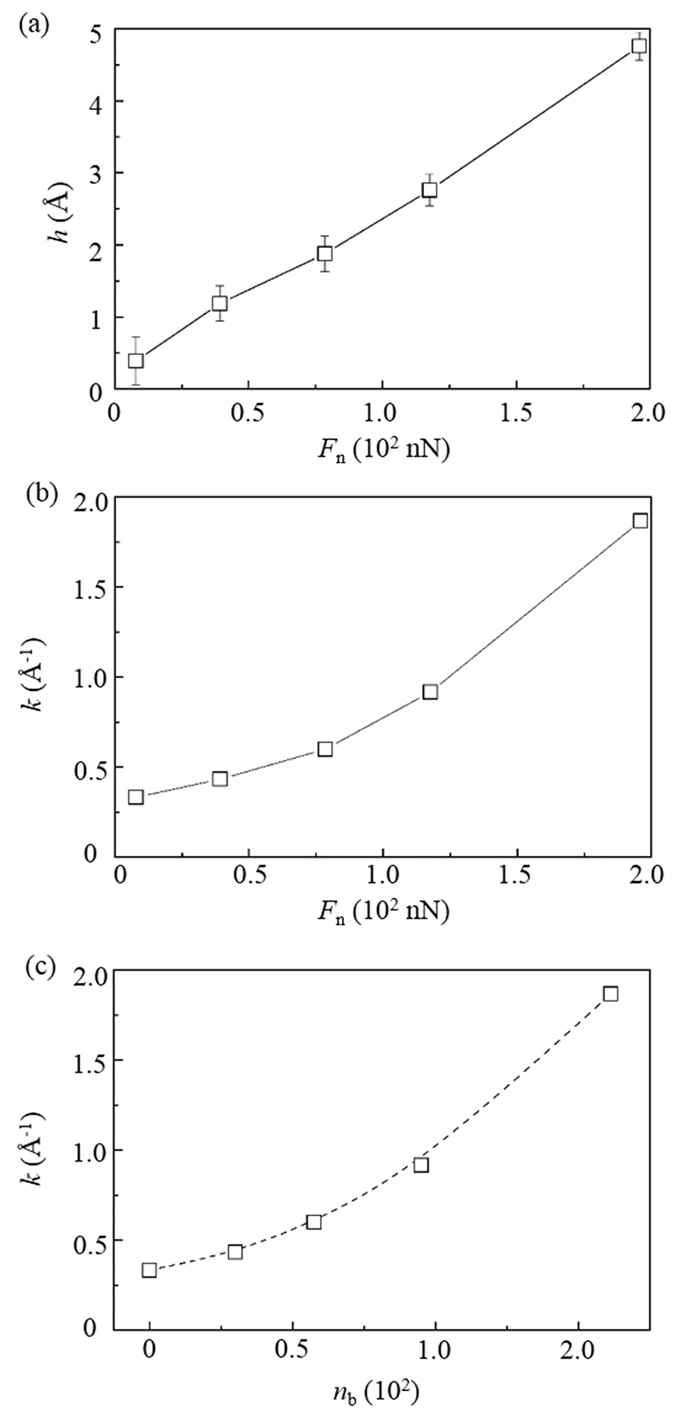
Effect of load *F*_n_ of the DLC film on (**a**) its wear rate *k* and (**b**) the sliding depth of the spherical particle *h*; (**c**) *k* vs *h*.

At a large *F*_n_, the spherical particle highly penetrates into DLC films, which can be verified by the increase of the sliding depth of the spherical particle (Fig. 3a). As a result, the large deformations of the DLC films can be caused by the spherical particle and highly resist their sliding, thus increasing the *F*_f_. This keeps consistent with results in the literature^14^. It has been reported that the rolling friction can be highly generated by the energy dissipation involved in the deformation of materials^15^. Sun *et al*. further found that the plowing is a significant factor in determining the three-body friction and wear within the elastic-plastic regime^10^. Bhushan *et al*. also reported that the three-body friction is caused by both the adhesion and plastic deformation which represents the plowing^16^. Therefore, it is evident that the nonlinear relation between the *F*_f_ and *n*_b_ at a large *F*_n_ is caused by the plastic deformation and plowing of the DLC films.

Moreover, it is noticed that the *F*_f_ is still high when *F*_n_ = 0. The high *F*_f_ is due to the presence of interfacial adhesion at the zero *F*_n_ and indicates that the *F*_f_ in the three-body contact condition has a direct proportion with the *n*_b_ instead of *F*_n_. This keeps consistent with observations in the previous studies under the two-body contact condition^12^,^13^.

The load *F*_n_ also significantly influences the wear performance of DLC films, as shown in Fig. 3b. The wear rate *k* increases with the *F*_n_. Since wear induced by adhesion is proportional to the real contact area that can be represented by *n*_b_, the relation between the *k* and *n*_b_ is meaningful in the investigation of adhesive wear. Figure 3c shows that the relation can be regarded as linear at a small *n*_b_ but becomes nonlinear when the *n*_b_ is large. The linear relation is due to the fact that the wear at a small *F*_n_ is determined by the interfacial adhesion while the nonlinear relation is attributed to the plastic deformation of the DLC films when the *F*_n_ is large.

It is noticed that *k* is nonzero when *F*_n_ = 0. Such nonzero *k* is different from results in the literature. Zhang *et al*. reported that a neglected wear can be obtained at a small load that only induces elastic deformation of material surface^9^. This neglected wear should be due to the von der Waals interactions at the sliding interface^9^. In the present study, however, the interfacial forces caused by the strong covalent C-C bonds are quite high and thus can induce worn atoms even when *F*_n_ = 0.

The contribution of the deformation of DLC films to their wear can be examined by analyzing the sliding depth *h* of the particle. Figure 3a shows that at the maximum *F*_n_ the *h* approaches to 5 Å which is higher than the displacement criteria for the definition of worn atoms. Because the surface of the DLC films deforms locally, many atoms are worn by such huge deformation even without the sliding. This keeps consistent with the worn atoms caused by plastic deformation in the literature^14^,^16^,^17^.

It is noticed that the wear of DLC films caused by their deformations is neglected when the *F*_n_ is small. In this case, the wear is determined by adhesion and can be predicted according to the proportion between *k* and *n*_b_. Since this proportion is commonly obtained in the two-body contact conditions^18^, its validation in this study indicates it represents the essence of the adhesion wear regardless of in the two-body or three-body contact conditions.

### Velocity effect

**Figure 4:**
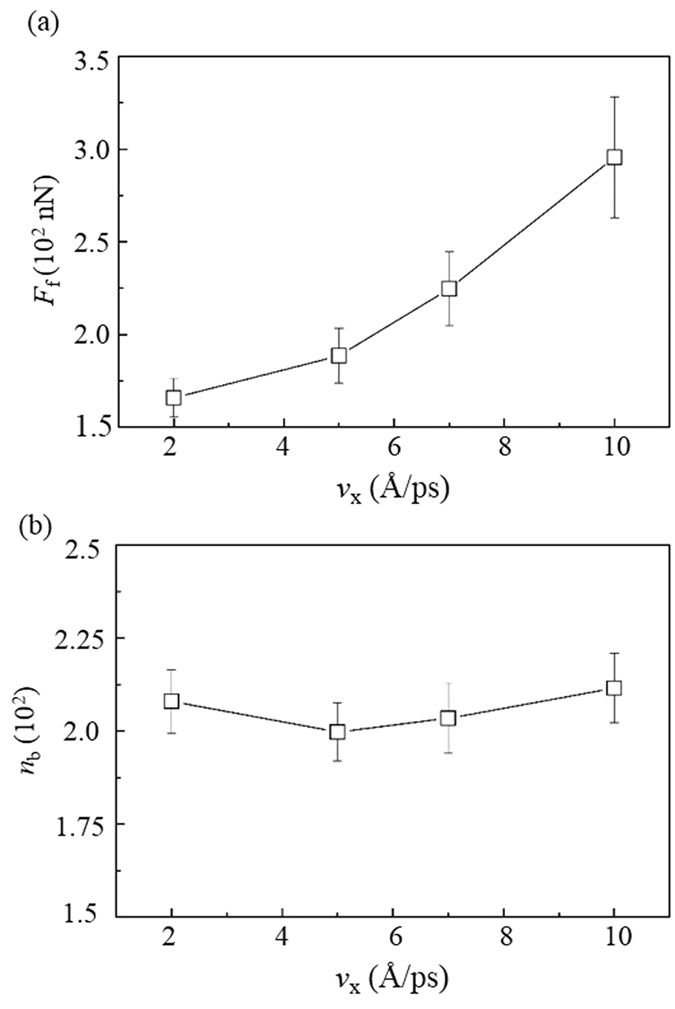
Effect of velocities *v*_x_ of the DLC film on (**a**) its friction force *F*_f_ and (**b**) the number of contact bonds *n*_b_.

Besides the load, the velocity *v*_x_ also highly influences the friction and wear of DLC films, as shown in Fig. 4a. The friction force *F*_f_ increases with the *v*_x_. This is different from the observations in the two-body contact conditions. It has been observed that the *F*_f_ commonly decreases with the *v*_x_ due to the fact that a high *v*_x_ can largely increase the friction temperature and reduce the *n*_b_^19^. However, in the present study the *n*_b_ almost keeps constant when the *v*_x_ varies (Fig. 4b). This constant *n*_b_ seems to conflict with the increase of *F*_f_, indicating the existence of a unique friction mechanism.

**Figure 5:**
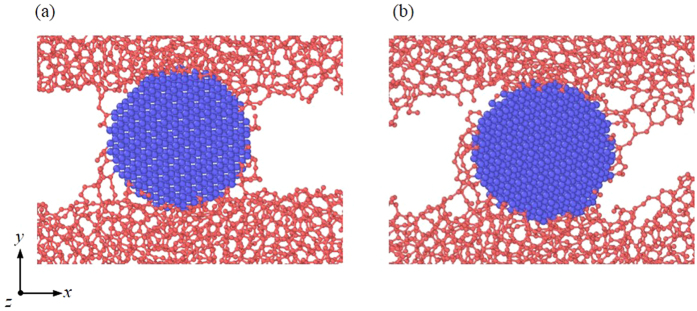
Atomic configuration with different velocities *v*_x_: (**a**) 2 Å/ps and (**b**) 10 Å/ps.

The friction mechanism can be understood by analyzing the friction configurations, as shown in Fig. 5. When the *v*_x_ increases, although the *n*_b_ is constant, many networks of DLC films attach to the particle. These networks can highly resist the sliding of the DLC film and thus largely increase the *F*_f_. The attaching of these networks to the particle is due to the structural response of DLC films to *v*_x_. With a high *v*_x_ which indicates a high strain rate, the micro-cracks in the DLC films have insufficient time to be initiated and propagate, thus increasing their yield strains. This keeps consistent with the mechanical theory of solids^20^,^21^,^22^ and has also been proved by our tensile simulations of the DLC films. For these films in the present study, such high strain is also attributed to the high flexibility of DLC networks at a high *v*_x_, which will be discussed later. As a result, more networks can attach to the third particle at a high *v*_x_, leading to the increase of *F*_f_.

**Figure 6:**
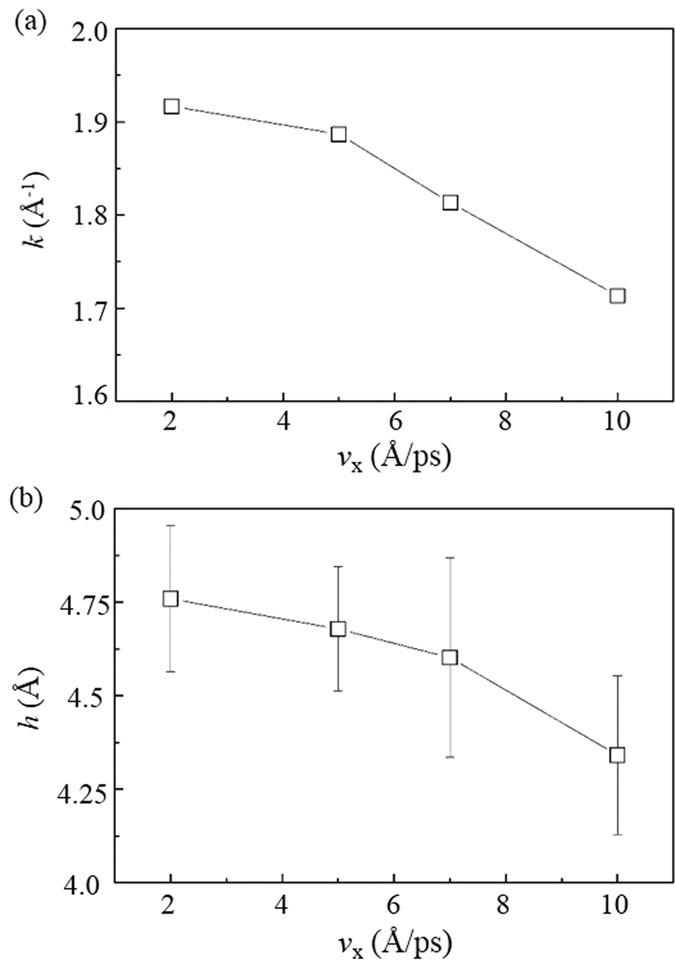
Effect of velocities *v*_x_ of the DLC film on (**a**) its wear rate *k* and (**b**) the sliding depth *h* of the spherical particle.

The wear performances of DLC films are also influenced by the *v*_x_, as shown in Fig. 6a. The wear rate *k* decreases with the *v*_x_. This trend agrees well with the decrease of the sliding depth *h* at a high *v*_x_ (Fig. 6b). The decrease of the *h* indicates the reduced deformation of the DLC films and thus contributes to the decrease of *k* by highly reducing plowing.

**Figure 7:**
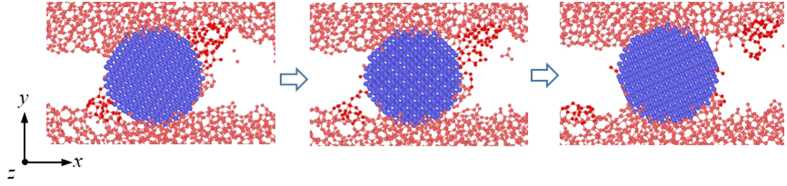
Formation process of worn atoms at a high velocity *v*_x_ of 10 Å/ps. The dark-red color in the figure highlights the networks attached to the third particle.

Effect of *v*_x_ on the adhesive wear also contributes to the reduction of *k*. Figure 7 shows that at a high *v*_x_ many DLC networks attach to the spherical particle. The relative sliding between the DLC films largely deform their networks. When the strain energy is high enough to break the bonds between these networks and the spherical particle, majority of them return to the DLC films while only few atoms are worn and still attach to the particle.

**Figure 8:**
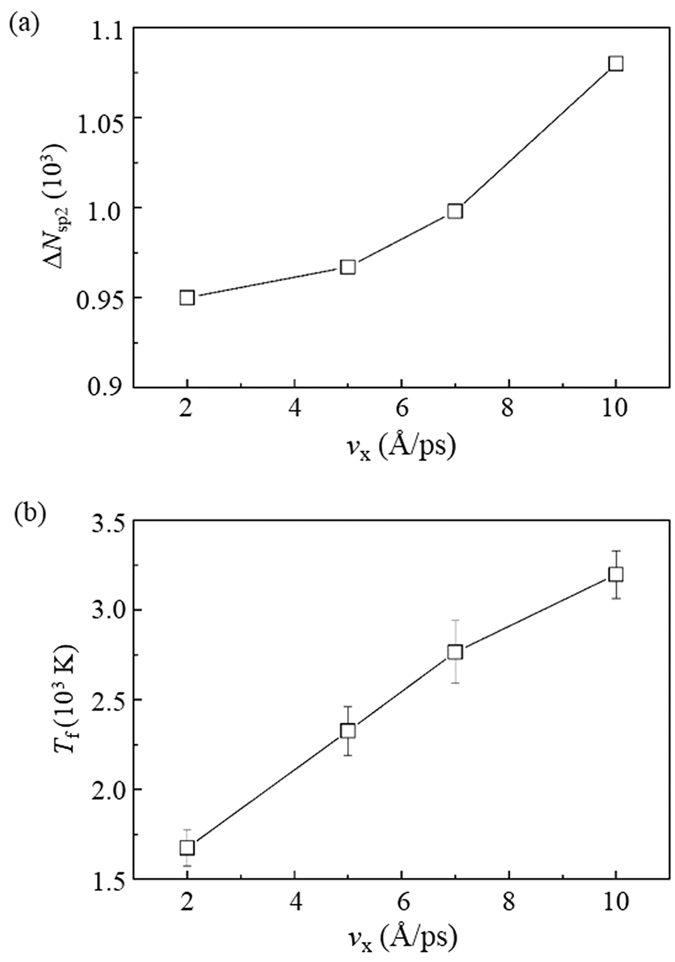
Effect of velocities *v*_x_ of the DLC film on its (**a**) number of new sp^2^ atoms Δ*N*_sp2_ and (**b**) friction temperature *T*_f_.

The return of the networks is determined by their flexibility and the instability of their atoms attached to the spherical particle. The flexibility of the DLC networks can be characterized by the fraction of sp^2^ atoms. This fraction can be represented by the total number of new sp^2^ atoms Δ*N*_sp2_ in the friction process. A higher Δ*N*_sp2_ indicates a larger fraction of sp^2^ atoms in the DLC film. Figure 8a shows that the Δ*N*_sp2_ increases with the *v*_x_, i.e., the DLC film exhibit more flexible behavior at a high *v*_x_. Such flexibility makes the DLC networks undergo high strain before their yielding.

On the other hand, the instability of the network atoms attached to the spherical particle can be simply characterized by their temperature which can be regarded as the friction temperature *T*_f_. The atoms with a high *T*_f_ are active and easily influenced by external forces. Figure 8b shows that the *T*_f_ highly increases with the *v*_x_.

The combination of the flexibility of the DLC networks and the high *T*_f_ can raise a new wear mechanism. When the DLC films relatively slide, their flexible networks can largely attach to the spherical particle and deform during the sliding (Fig. 7a,b). As a result, such networks can highly draw the atoms bonded to the particle and make them tend to return to the DLC films. Moreover, the tendency is further enhanced by the high *T*_f_, since it can highly improve the possibility of the breaks for the bonds formed between these atoms and the particle. As a result, majority of these atoms return to the DLC film and only few of them are worn, resulting in the decrease of *k*.

The tribological mechanisms of DLC films with different *v*_x_ in this study are quite different from results under the two-body contact condition in the literature^19^,^23^,^24^,^25^. It has been reported that the high *v*_x_ can reduce the friction and wear of DLC films by improving their level of graphitization and promoting the formation of a transfer film which is easy to shear and capable of isolating the DLC films from their counterparts.

The present study shows that although DLC networks attaching to the third particle are actually the transfer layer, the friction reduction by such layer is neglected. This is attributed to that the friction reduction by the transfer layers is caused by their shear deformation due to their easy-shear properties. However, in this study their shear deformations hardly occur because the pure rolling of the spherical particle mainly causes tensile deformation of the DLC networks.

Therefore, the present study generally shows that the wear reduction at a high *v*_x_ in the three-body contact conditions is caused not by the formation of transfer layer but by suppressing the plowing and improving the flexibility of the DLC networks and the high friction temperature.

### Particle effect

The effect of the particle size is considered by changing the radius of the spherical particle, as shown in Table 1. The friction force *F*_f_ increases with a small-sized particle. Such increase is attributed to that under the same load the small-sized particle causes a high contact stress and thus largely penetrates into the DLC films. Such penetration highly deforms the DLC films and thus increases the *F*_f_. This keeps consistent with results in the literature which stated that the plowing is significant in determining the three-body friction at the nanoscale^10^,^26^. The large deformation of the DLC films with the small-sized particle also increases their wear rate.

The large *F*_f_ and *k* of DLC films with a small-sized particle is different from observations in the previous studies^2^,^5^,^6^,^8^. Qi *et al*. reported that small-sized sand particles can reduce the friction and wear of DLC films mainly by reducing the contact stress^5^,^6^. This is because in their studies a large quantity of sand particles are located at the interfaces between DLC films. The small size of such particles can make them form a relatively flat layer between the DLC films and thus reduce the contact stress. However, the present study demonstrates that a small number of particles surely exhibit a different abrasive behavior and may severely damage the DLC film and change their tribological behaviors.

**Figure 9:**
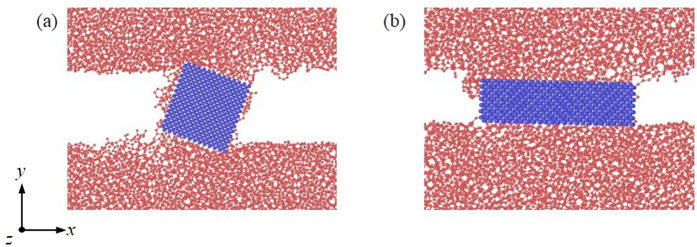
Atomic configuration with (**a**) cubic and (**b**) cuboidal particles during the friction process.

The particle shape can also influence the tribological behaviors of DLC films. Figure 9 shows that the cubic particle also purely rolls when the DLC films relatively slide. However, the cuboid particle hardly rolls and is initially attached to the lower DLC film. Such cuboid particle highly ploughs the upper DLC film. This can be proved by the chip formation in front of the cuboid particle. Moreover, the chip exerts a high force to the cuboid particle. When this chip becomes large, this force plus the adhesion force from the upper DLC film can cause the movement of the cuboid particle. As a result, such particle is in turn attached to the upper DLC film and ploughs the lower DLC film. It can be seen that the shape of particles highly influences their movement modes which affect tribological mechanisms of DLC films. This keeps consistent with observations in the previous study which stated that the nanoparticle exhibits an optimum shape to realize its rolling^27^.

**Figure 10:**
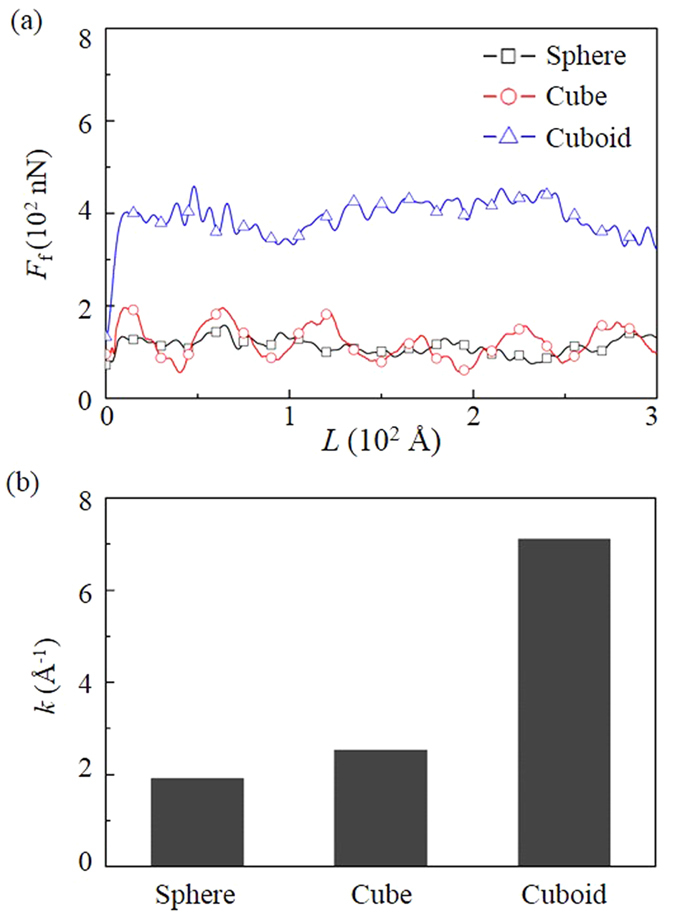
Effect of the shape of the third particle on the friction and wear of DLC films.

Figure 10a shows that the cuboid particle causes a high *F*_f_ while the spherical and cubic particles cause a low *F*_f_. The dependence of *F*_f_ on the particle shape is due to the transition of the friction mechanisms. For the cuboid particle, the ploughing is present and highly increases the *F*_f_. For the spherical and cubic particles, the friction is determined by rolling and the *F*_f_ is caused by the adhesion instead of the ploughing. The variance of the *F*_f_ for cubic particle is due to the varied interfacial adhesion strength when the particle rolls. Therefore, it can be seen that the presence of ploughing is a significant reason for the high friction in the three-body contact condition.

The shape of the third particle also highly influences the wear performance of DLC films (Fig. 10b). The *k* with the cuboid particle is much higher than those with the cubic and spherical particles and is attributed to the occurrence of ploughing. Meanwhile, the low *k* with cubic and spherical particles is due to the rolling adhesive wear which is highly suppressed by the flexibility of the DLC networks.

The influence of the particle shape indicates that the presence of ploughing can highly increase the friction and wear of DLC films. Moreover, such influence demonstrates that the rolling wear rate of the DLC films in the third-body contact condition is lower than their sliding wear rate. This is consistent with results in the previous studies^10^,^28^. It has been reported that plowing or ploughing dominates the three-body wear at the nanoscale^10^. Experimental results also found that grooving movement of particles shows a higher contribution to wear volume of workpiece than their rolling mode^28^. The ploughing caused by the cuboidal particle actually reflects the properties of the two-body abrasion which cannot be well explained by the mechanisms for spherical and cubic particles. Therefore, it is evident that the shape of the particle can directly determine its movement mode and the friction and wear mechanisms of the DLC films^27^.

## Conclusions

The tribological behaviors of DLC films with a third particle at their contact interface are investigated via molecular dynamics simulations. The influence factors such as the load *F*_n_, velocity *v*_x_, shape of the particle and its size are considered. It has been found that the friction force *F*_f_ and wear rate *k* of the DLC film are determined by adhesion at a small *F*_n_ but dominated by both adhesion and plowing at a large *F*_n_. This can be verified by examining the relation of the *F*_f_ and *k* with the number of bonds *n*_b_ at the contact interface. With the increase of *v*_x_, the *F*_f_ increases and *k* decreases while the *n*_b_ almost keeps constant. This is because with a large *v*_x_ the DLC networks exhibit a large yield strain and thus largely attach to the third particle to resist the relative sliding of the DLC films. These attached networks highly increase the *F*_f_. The decrease of *k* at a large *v*_x_ is caused by the flexibility of the DLC networks and the decrease of the sliding depth. The small-sized particle can increase the *F*_f_ and *k* by enhancing plowing. The shape of the third particle can highly influence its movement mode and change the friction and wear of the DLC films. It is found that the spherical and cubic particle purely roll without sliding. However, the cuboidal particle highly increases the *F*_f_ and *k* by purely sliding and ploughing the DLC films, indicating that the cuboidal particle can induce the transition of tribological mechanisms from a three-body rolling to a two-body sliding. Note that in this study the third particles are set to be rigid and thus their wear are not considered. However, such zero-wear particles are uncommon in experiments. Future research will consider the wear properties of compliant particles in a real sand-dust problem and explore their influences on the tribological behaviors of DLC films.

## Methods

**Figure 11:**
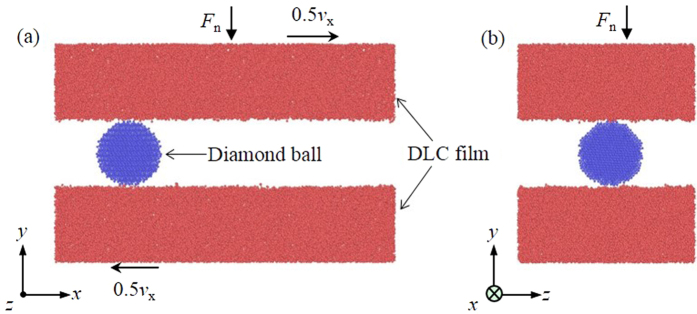
Atomic configuration of the simulation model with (**a**) a front view and (**b**) a side view. The model consists of two relative sliding DLC films with a third particle located between them.

The simulation system consists of two relatively sliding DLC films and a particle located at the interface between them (Fig. 11). The relative sliding is realized by setting the upper and lower DLC film with a velocity of 0.5*v*_x_ and −0.5*v*_x_ along the *x*-direction, respectively. During the sliding, a load is applied by maintaining a force *F*_n_ to the upper DLC film along the *y*-direction. The periodic boundary conditions of the system are set along its *x* and *z*-directions.

The particle has a diamond crystalline structure and is set as a rigid body to avoid its wear during the simulation. The spherical, cubic and cuboidal particles are generated to study the effect of their shapes. The spherical particle has a radius of 12 Å. The cube and cuboid particle have dimensions of 20 × 20 × 20 Å^3^ and 40 × 15 × 20 Å^3^, respectively. A small spherical particle with a radius of 10 Å is also generated to study its size effect.

The DLC films have dimensions of 120 × 25 × 60 Å^3^. Each of the DLC films is defined into three different layers from the contact interface to subsurface along the *y*-direction: Newtonian, thermostatic and rigid layers. The Newtonian layer with a thickness of 18 Å is in contact with the diamond ball and contains atoms that are free to move under the forces of their neighbors. The thermostat layer with a thickness of 3 Å is employed keep a constant temperature of 300 K by rescaling the velocities of atoms. The rigid layer with a thickness of 4 Å can help to maintain velocity. Moreover, the rigid layer in the upper DLC film is set to take *F*_n_ along the *y*-direction while that in the lower DLC film is prohibited to move along the same direction. More details of the simulation model can refer to our previous studies^19^,^29^.

The DLC films in the simulations are obtained by a melt-quenching procedure^30^. A block of crystalline diamond is firstly generated. In a canonical NVT ensemble, the temperature of such block is raised to be above the melting point of crystalline diamond, thus leading to the formation of carbon liquid. After a period of equilibration by keeping the block thermostatic, its temperature decreases to 300 K with a high rate of about 1000 K/ps which allows proper structural relaxations in amorphous structures. The dimension of the block is finally adjusted to release its residual stress in an isothermal-isobaric NPT ensemble at 300 K. More details of the melt-quenching procedure can refer to previous studies^30^,^31^.

The simulations are conducted via the large-scale atomic/molecular massively parallel simulator (LAMMPS)^32^. The atomic interactions are described by the Tersoff potential which is capable of studying the structures and energetics of carbon-based materials^33^,^34^. Moreover, the time step of the simulations is set as 1 fs, and their molecular visualizations are conducted via the software OVITO^35^.

Prior to the friction simulation, a load *F*_n_ (7.8, 39.2, 78.4, 117.6 and 196 nN) is applied to the upper DLC film to cause contact between the particle and the DLC films. The contact is equilibrated within 30 ps. After the equilibration period, the DLC films start to relatively slide and the relative velocity *v*_x_ is set as 2, 5, 7 and 10 Å/ps, respectively. The total sliding distance is always kept as 300 Å. In the cases with different *F*_n_, *v*_x_ is kept as 2 Å/ps. The *F*_n_ is set as 196 nN for the cases with various *v*_x_. In the cases with different shapes and sizes of particles, the *F*_n_ and *v*_x_ are set as 196 nN and 2 Å/ps, respectively.

The friction force *F*_f_ is calculated by summing up the forces of atoms in the rigid layer of the upper DLC film along the *x*-direction. The number of worn atoms *N* is simply calculated by evaluating their displacements^17^. For a mild wear which is determined by atom-by-atom attritions^36^, a worn atom can be conveniently defined as the one whose bonds with its nearest neighbors break during the friction process, Such breaks can be caused when the displacement of the atom is larger than two-bond length. Since the maximum length of a C-C bond in the DLC films is about 2 Å which corresponds to the first minimum in their radial distribution functions, the length of 4 Å can be chosen as the displacement criteria to estimate the worn atoms. This criteria is also useful to estimate the *N* for the severe wear of materials that is determined by their plastic deformations. Previous studies calculated this *N* as those removed from the wear track^37^,^38^. It is evident that these atoms have displacements larger than 4 Å. Therefore, the present criteria can provide more information of wear than the method employed in the literature^37^,^38^. It should be noticed that the large displacement of atoms in the DLC films can also be induced by their elastic deformation. These elasticity-induced displacements can cause an error in the wear calculation. In order to eliminate such error, the wear calculation is conducted after the friction sliding. In this case, the elastic deformation has recovered, and thus all the large displacements of atoms are caused by the plastic deformation of the DLC films.

The wear rate *k* is calculated as *k* = *N*/*L*, where *N* is the number of worn atoms and *L* is the sliding distance. The average *k* of two DLC films is employed to indicate their wear performance. It is noticed that the sliding tracks overlap in each simulation due to the periodic condition in the x-direction. The overlapping hardly changes the wear rate and the friction force, thus indicating that this study actually investigates the phenomenon in the running-in period of wear tests in experiments. The hybridization states of C atoms are also evaluated by calculating the number of their nearest neighbor atoms within the cutoff of the maximum bond length. The fourfold, threefold and twofold atoms are regarded as sp^3^, sp^2^ and sp bonded, respectively^30^. The temperature of atoms is calculated based on its relation with their kinetic energies^39^.

## Additional Information

**How to cite this article**: Bai, L. *et al*. Influence of Third Particle on the Tribological Behaviors of Diamond-like Carbon Films. *Sci. Rep.* **6**, 38279; doi: 10.1038/srep38279 (2016).

**Publisher's note:** Springer Nature remains neutral with regard to jurisdictional claims in published maps and institutional affiliations.

## Figures and Tables

**Table 1:** Effect of particle radius *r* on the friction and wear of DLC films.

	*F*_f_ (nN)	*k* (Å^−1^)
*r* = 10 Å	204	2.67
*r* = 12 Å	181	1.92
